# (2,2′-Bipyridine-κ^2^ *N*,*N*′)chlorido(1,4,7-trithia­cyclo­nonane-κ^3^ *S*,*S*′,*S*′′)ruthenium(II) nitrate monohydrate

**DOI:** 10.1107/S1600536810046556

**Published:** 2010-11-13

**Authors:** José A. Fernandes, Filipe A. Almeida Paz, Maria João P. Mota, Susana S. Braga, Teresa M. Santos

**Affiliations:** a Department of Chemistry, University of Aveiro, CICECO, 3810-193 Aveiro, Portugal

## Abstract

In the title compound, [RuCl(C_10_H_8_N_2_)(C_6_H_12_S_3_)]NO_3_·H_2_O or [RuCl(bpy)([9]aneS_3_)]NO_3_·H_2_O, ([9]aneS_3_ is 1,4,7-tri­thia­cyclo­nonane and bpy is 2,2′-bipyridine), the Ru^II^ cation has a slightly distorted octa­hedral environment composed of three facially coordinated S atoms from ([9]aneS_3_), two N atoms from bpy and a chloride anion. The nitrate counter-ion and the water mol­ecule of crystallization are engaged in O—H⋯O hydrogen-bonding inter­actions, leading to a supra­molecular chain running parallel to the *c* axis.

## Related literature

For general background on the cytotoxic activity of com­pounds with the (Ru[9]aneS_3_) unit, see: Bratsos *et al.* (2008 ▶); Serli *et al.* (2005 ▶). For related compounds, see: Sala *et al.* (2004 ▶); Marques, Braga *et al.* (2009 ▶); Marques, Santos *et al.* (2009 ▶); Marques *et al.* (2008 ▶). For compounds with the same cation as the title compound, see: Serli *et al.* (2005 ▶); Goodfellow *et al.* (1997 ▶). For graph-set notation for hydrogen-bonded aggregates, see: Grell *et al.* (1999 ▶)
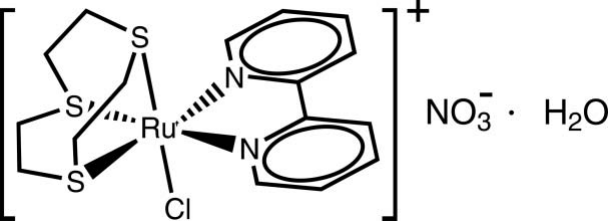


## Experimental

### 

#### Crystal data

[RuCl(C_10_H_8_N_2_)(C_6_H_12_S_3_)]NO_3_·H_2_O
*M* *_r_* = 553.07
Monoclinic, 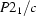

*a* = 7.6523 (4) Å
*b* = 25.1887 (12) Å
*c* = 11.1099 (5) Å
β = 108.438 (2)°
*V* = 2031.52 (17) Å^3^
*Z* = 4
Mo *K*α radiation
μ = 1.24 mm^−1^
*T* = 150 K
0.05 × 0.04 × 0.02 mm

#### Data collection

Bruker X8 Kappa CCD APEXII diffractometer
Absorption correction: multi-scan (*SADABS*; Sheldrick, 1998 ▶) *T*_min_ = 0.941, *T*_max_ = 0.976
21232 measured reflections
5360 independent reflections
4179 reflections with *I* > 2σ(*I*)
*R*_int_ = 0.048

#### Refinement

*R*[*F* ^2^ > 2σ(*F* ^2^)] = 0.045
*wR*(*F* ^2^) = 0.078
*S* = 1.08
5360 reflections
259 parameters
3 restraints
H atoms treated by a mixture of independent and constrained refinement
Δρ_max_ = 0.74 e Å^−3^
Δρ_min_ = −0.60 e Å^−3^

### 

Data collection: *APEX2* (Bruker, 2006 ▶); cell refinement: *SAINT-Plus* (Bruker, 2005 ▶); data reduction: *SAINT-Plus*; program(s) used to solve structure: *SHELXTL* (Sheldrick, 2008 ▶); program(s) used to refine structure: *SHELXTL*; molecular graphics: *DIAMOND* (Brandenburg, 2009 ▶); software used to prepare material for publication: *SHELXTL*.

## Supplementary Material

Crystal structure: contains datablocks global, I. DOI: 10.1107/S1600536810046556/bg2373sup1.cif

Structure factors: contains datablocks I. DOI: 10.1107/S1600536810046556/bg2373Isup2.hkl

Additional supplementary materials:  crystallographic information; 3D view; checkCIF report

## Figures and Tables

**Table 1:** Hydrogen-bond geometry (Å, °)

*D*—H⋯*A*	*D*—H	H⋯*A*	*D*⋯*A*	*D*—H⋯*A*
O1*W*—H1*X*⋯O3^i^	0.95 (3)	2.10 (3)	3.037 (4)	169 (4)
O1*W*—H1*Y*⋯O2^ii^	0.94 (3)	1.92 (2)	2.839 (4)	166 (4)

Symmetry codes: (i) 
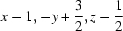
; (ii) 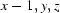.

**Fig. 1.**
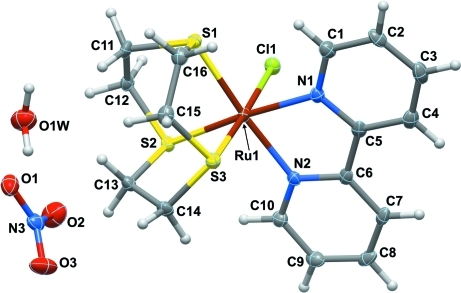
Asymmetric unit of the title compound. Non-hydrogen atoms are represented as thermal ellipsoids drawn at the 50% probability level while hydrogen atoms are drawn as small spheres with arbitrary radii. The labelling scheme is provided for all non-hydrogen atoms.

**Fig. 2.**
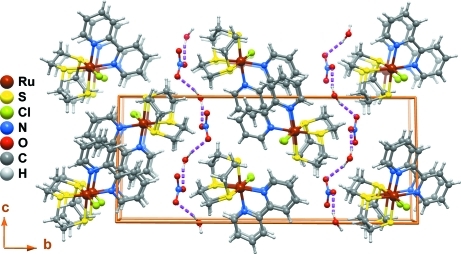
Crystal packing of the title compound viewed in perspective along the a axis. The C22 chain is highlighted in dashed pink lines. For geometrical details on the represented hydrogen bonding interactions see Table 1.

## supplementary crystallographic information

### Comment

The ruthenium coordination complexes containing 1,4,7-trithiacyclonane ([9]aneS_3_) have been studied for their activity as antitumoral agents (Bratsos *et al.*, 2008; Serli *et al.*, 2005). Some members of this family of compounds have been tested as cytotoxic (Marques, Santos *et al.*, 2009) and antimicrobial (Marques, Braga *et al.*, 2009) agents, or as catalysts (Sala *et al.*, 2004). We have also studied the solid-state properties of the inclusion compounds of a number of these ruthenium compounds in cyclodextrins (Marques *et al.*, 2008). While reacting the Ru^II^ precursor [Ru([9]aneS_3_)(bpy)Cl]Cl with AgNO_3_ we isolated the title compound as a secondary product.

The asymmetric unit of the title compound (see Scheme) comprises a whole [Ru([9]aneS_3_)(bpy)Cl]^+^ cation, a charge-balancing nitrate anion and a water molecule of crystallization (Figure 1). A survey in the Cambridge Structural Database revealed that this Ru^II^ cation has already been described by other groups while co-crystallizing with different anions, namely chloride and trifluoromethanesulfonate (Serli *et al.*, 2005; Goodfellow *et al.*, 1997). This cation has an octahedral coordination environment for Ru^II^ with the tricoordinating [9]aneS_3_ molecule occupying one of the faces of the polyhedron [Ru—S distances ranging from 2.2800 (9) to 2.4379 (8) Å]. The remaining three coordination sites are occupied by a chelating 2,2'-bipyridine (bpy) [Ru—N distances of 2.088 (3) and 2.093 (3) Å], and a chlorido anion [Ru—Cl distance of 2.4379 (8) Å]. The octahedral angles of the coordination polyhedron fall within a short range of the ideal values: while the *cis* angles range from 77.86 (10) to 98.04 (7)°, the *trans* angles are in the 174.02 (8)–177.46 (3)° range.

The water molecule of crystallization and the charge-balancing nitrate anion interact *via* strong hydrogen bonds (Table 1), leading to a polymeric H1Y—{O1W—H1X···O3—N3—O2···H1Y}_∞_ chain running parallel to the *c*-axis (dashed pink lines in Figure 2), which can be described by a *C^2^_2_* graph set motif (Grell *et al.*, 1999). Noteworthy, these hydrogen bonds are of strong nature [O···O distances of 3.037 (4) and 2.839 (4) Å] and highly directional [O—H···O angles of 169 (4) and 166 (4)°].

The title compound, based on the [Ru([9]aneS_3_)(bpy)Cl]^+^ cation, is considerably different from the two previously related structures: while in our structure the charge-balancing nitrate co-crystallizes with one water molecule, the chloride and trifluoromethanesulfonate structures contain three and none of these entities, respectively (Goodfellow *et al.*, 1997; Serli *et al.*, 2005). Noteworthy, in the former structure (having three water molecules) the crystal packing exhibits a supramolecular two-dimensional network of hydrogen bonding interactions. In the title compound the presence of a single water molecule in the composition promotes the formation of only a one-dimensional supramolecular chain (see above). In summary, it is feasible to consider the title compound as an intermediary case between the two already known structures.

### Experimental

The Ru^II^ precursor [Ru([9]aneS_3_)(bpy)Cl]Cl was prepared according to reported methods (Goodfellow *et al.*, 1997). Remaining chemicals were purchased from commercial sources and used as received without further purification. [Ru([9]aneS_3_)(bpy)Cl]Cl (105.6 mg; 0.21 mmol) was treated with AgNO_3_ (53.0 mg; 0.31 mmol) in order to produce an intermediate labile species by exchanging the coordinated Cl^-^ ligand by a solvent molecule (20 minutes stirring at room temperature in 50 ml of commercial grade ethanol). A white precipitate (AgCl) was filtered off through Celite, and the volume of the remaining red-orange solution was reduced to half by evaporation at 50 °C in a rotatory evaporator. After one month at -18 °C, the title compound was isolated as orange crystals.

### Refinement

Hydrogen atoms bound to carbon were located at their idealized positions and were included in the final structural model in riding approximation with C—H = 0.95 Å (aromatic C—H) and 0.99 Å (—CH_2_). The isotropic thermal displacement parameters for these atoms were fixed at 1.2 times *U*_eq_ of the respective parent carbon atom.

Hydrogen atoms associated with the water molecule of crystallization have been directly located from difference Fourier maps and were included in the final structural model with the distances restrained to 0.95 (1) Å and *U*_iso_*=1.5×U*_eq_ of the respective parent oxygen atom.

### Crystal data

**Table:**

[RuCl(C_10_H_8_N_2_)(C_6_H_12_S_3_)]NO_3_·H_2_O	*F*(000) = 1120
*M**_r_* = 553.07	*D*_x_ = 1.808 Mg m^−^^3^
Monoclinic, *P*2_1_/*c*	Mo *K*α radiation, λ = 0.71073 Å
Hall symbol: -P 2ybc	Cell parameters from 5581 reflections
*a* = 7.6523 (4) Å	θ = 2.5–30.4°
*b* = 25.1887 (12) Å	µ = 1.24 mm^−^^1^
*c* = 11.1099 (5) Å	*T* = 150 K
β = 108.438 (2)°	Block, orange
*V* = 2031.52 (17) Å^3^	0.05 × 0.04 × 0.02 mm
*Z* = 4	

### Data collection

**Table:**

Bruker X8 Kappa CCD APEXII diffractometer	5360 independent reflections
Radiation source: fine-focus sealed tube	4179 reflections with *I* > 2σ(*I*)
graphite	*R*_int_ = 0.048
ω and φ scans	θ_max_ = 29.1°, θ_min_ = 3.7°
Absorption correction: multi-scan (*SADABS*; Sheldrick, 1998)	*h* = −10→9
*T*_min_ = 0.941, *T*_max_ = 0.976	*k* = −34→29
21232 measured reflections	*l* = −15→15

### Refinement

**Table:**

Refinement on *F*^2^	Primary atom site location: structure-invariant direct methods
Least-squares matrix: full	Secondary atom site location: difference Fourier map
*R*[*F*^2^ > 2σ(*F*^2^)] = 0.045	Hydrogen site location: inferred from neighbouring sites
*wR*(*F*^2^) = 0.078	H atoms treated by a mixture of independent and constrained refinement
*S* = 1.08	*w* = 1/[σ^2^(*F*_o_^2^) + (0.026*P*)^2^ + 1.766*P*] where *P* = (*F*_o_^2^ + 2*F*_c_^2^)/3
5360 reflections	(Δ/σ)_max_ = 0.002
259 parameters	Δρ_max_ = 0.74 e Å^−^^3^
3 restraints	Δρ_min_ = −0.60 e Å^−^^3^

### Special details

**Table:**

Geometry. All e.s.d.'s (except the e.s.d. in the dihedral angle between two l.s. planes) are estimated using the full covariance matrix. The cell e.s.d.'s are taken into account individually in the estimation of e.s.d.'s in distances, angles and torsion angles; correlations between e.s.d.'s in cell parameters are only used when they are defined by crystal symmetry. An approximate (isotropic) treatment of cell e.s.d.'s is used for estimating e.s.d.'s involving l.s. planes.
Refinement. Refinement of *F*^2^ against ALL reflections. The weighted *R*-factor *wR* and goodness of fit *S* are based on *F*^2^, conventional *R*-factors *R* are based on *F*, with *F* set to zero for negative *F*^2^. The threshold expression of *F*^2^ > σ(*F*^2^) is used only for calculating *R*-factors(gt) *etc*. and is not relevant to the choice of reflections for refinement. *R*-factors based on *F*^2^ are statistically about twice as large as those based on *F*, and *R*- factors based on ALL data will be even larger.

### Fractional atomic coordinates and isotropic or equivalent isotropic displacement parameters (Å^2^)

**Table:**

	*x*	*y*	*z*	*U*_iso_*/*U*_eq_	
Ru1	0.57746 (4)	0.910714 (10)	0.22233 (2)	0.01142 (7)	
S1	0.38460 (11)	0.90192 (3)	0.01591 (7)	0.01507 (17)	
S2	0.68086 (11)	0.82613 (3)	0.20539 (7)	0.01433 (17)	
S3	0.35078 (11)	0.87415 (3)	0.28829 (7)	0.01582 (17)	
Cl1	0.81988 (11)	0.94626 (3)	0.14642 (7)	0.01768 (17)	
N1	0.5087 (4)	0.98857 (10)	0.2537 (2)	0.0143 (6)	
N2	0.7509 (4)	0.92503 (10)	0.4070 (2)	0.0147 (6)	
N3	0.9073 (4)	0.70605 (11)	0.2395 (3)	0.0225 (7)	
O1	0.7828 (3)	0.70188 (10)	0.1357 (2)	0.0280 (6)	
O2	1.0661 (4)	0.71932 (12)	0.2423 (2)	0.0385 (7)	
O3	0.8748 (4)	0.69717 (11)	0.3408 (2)	0.0355 (7)	
C1	0.3886 (5)	1.02027 (13)	0.1688 (3)	0.0189 (7)	
H1	0.3243	1.0063	0.0874	0.023*	
C2	0.3555 (5)	1.07204 (13)	0.1954 (3)	0.0206 (7)	
H2	0.2682	1.0928	0.1335	0.025*	
C3	0.4493 (5)	1.09354 (14)	0.3120 (3)	0.0233 (8)	
H3	0.4284	1.1292	0.3317	0.028*	
C4	0.5749 (5)	1.06186 (14)	0.3996 (3)	0.0199 (7)	
H4	0.6424	1.0758	0.4804	0.024*	
C5	0.6021 (4)	1.00952 (13)	0.3691 (3)	0.0143 (7)	
C6	0.7357 (4)	0.97384 (13)	0.4554 (3)	0.0153 (7)	
C7	0.8451 (5)	0.98784 (14)	0.5772 (3)	0.0187 (7)	
H7	0.8325	1.0219	0.6104	0.022*	
C8	0.9720 (5)	0.95207 (15)	0.6495 (3)	0.0224 (8)	
H8	1.0458	0.9610	0.7332	0.027*	
C9	0.9903 (5)	0.90346 (14)	0.5989 (3)	0.0214 (8)	
H9	1.0786	0.8786	0.6464	0.026*	
C10	0.8781 (5)	0.89129 (14)	0.4777 (3)	0.0185 (7)	
H10	0.8917	0.8577	0.4430	0.022*	
C11	0.4255 (5)	0.83499 (13)	−0.0368 (3)	0.0179 (7)	
H11A	0.3333	0.8101	−0.0234	0.021*	
H11B	0.4098	0.8358	−0.1287	0.021*	
C12	0.6176 (5)	0.81530 (13)	0.0351 (3)	0.0172 (7)	
H12A	0.7076	0.8337	0.0024	0.021*	
H12B	0.6248	0.7769	0.0189	0.021*	
C13	0.5226 (5)	0.78117 (13)	0.2499 (3)	0.0169 (7)	
H13A	0.4228	0.7702	0.1727	0.020*	
H13B	0.5901	0.7489	0.2900	0.020*	
C14	0.4398 (5)	0.80803 (13)	0.3412 (3)	0.0179 (7)	
H14A	0.5350	0.8109	0.4255	0.022*	
H14B	0.3385	0.7857	0.3508	0.022*	
C15	0.1622 (4)	0.85625 (14)	0.1442 (3)	0.0191 (7)	
H15A	0.1780	0.8190	0.1211	0.023*	
H15B	0.0433	0.8589	0.1614	0.023*	
C16	0.1581 (4)	0.89212 (14)	0.0344 (3)	0.0189 (7)	
H16A	0.1075	0.9271	0.0472	0.023*	
H16B	0.0743	0.8767	−0.0448	0.023*	
O1W	0.1468 (4)	0.72516 (12)	0.0102 (3)	0.0371 (7)	
H1X	0.058 (4)	0.7458 (15)	−0.051 (3)	0.056*	
H1Y	0.106 (5)	0.7187 (17)	0.080 (2)	0.056*	

### Atomic displacement parameters (Å^2^)

**Table:**

	*U*^11^	*U*^22^	*U*^33^	*U*^12^	*U*^13^	*U*^23^
Ru1	0.01225 (13)	0.01040 (12)	0.01162 (11)	−0.00014 (11)	0.00379 (9)	−0.00145 (10)
S1	0.0154 (4)	0.0154 (4)	0.0138 (3)	0.0000 (3)	0.0038 (3)	−0.0017 (3)
S2	0.0147 (4)	0.0127 (4)	0.0161 (4)	−0.0002 (3)	0.0057 (3)	−0.0014 (3)
S3	0.0167 (4)	0.0155 (4)	0.0169 (4)	−0.0006 (4)	0.0077 (3)	−0.0015 (3)
Cl1	0.0169 (4)	0.0177 (4)	0.0201 (4)	−0.0022 (3)	0.0082 (3)	−0.0017 (3)
N1	0.0150 (14)	0.0133 (14)	0.0166 (12)	0.0005 (12)	0.0078 (11)	−0.0011 (10)
N2	0.0174 (15)	0.0122 (14)	0.0152 (12)	−0.0022 (12)	0.0062 (11)	0.0004 (10)
N3	0.0270 (18)	0.0167 (15)	0.0242 (15)	0.0051 (14)	0.0085 (13)	0.0028 (12)
O1	0.0273 (15)	0.0213 (14)	0.0292 (14)	0.0035 (12)	0.0002 (11)	−0.0024 (11)
O2	0.0268 (16)	0.0520 (19)	0.0373 (16)	−0.0106 (15)	0.0109 (12)	−0.0049 (14)
O3	0.0439 (18)	0.0402 (17)	0.0278 (14)	0.0133 (15)	0.0187 (13)	0.0132 (12)
C1	0.0197 (18)	0.0180 (18)	0.0209 (16)	0.0022 (15)	0.0094 (14)	0.0007 (13)
C2	0.0216 (19)	0.0142 (17)	0.0294 (18)	0.0067 (15)	0.0126 (15)	0.0052 (14)
C3	0.029 (2)	0.0152 (17)	0.0317 (18)	−0.0002 (17)	0.0179 (16)	−0.0038 (14)
C4	0.0220 (19)	0.0204 (18)	0.0211 (16)	−0.0023 (16)	0.0124 (14)	−0.0053 (14)
C5	0.0139 (16)	0.0161 (17)	0.0173 (15)	−0.0035 (14)	0.0114 (13)	−0.0020 (12)
C6	0.0155 (17)	0.0163 (17)	0.0163 (15)	−0.0073 (14)	0.0080 (13)	−0.0025 (12)
C7	0.0206 (18)	0.0195 (18)	0.0167 (15)	−0.0086 (15)	0.0078 (13)	−0.0065 (13)
C8	0.0209 (19)	0.031 (2)	0.0132 (15)	−0.0135 (17)	0.0025 (13)	−0.0018 (14)
C9	0.0181 (18)	0.0226 (19)	0.0202 (16)	−0.0038 (16)	0.0019 (13)	0.0050 (14)
C10	0.0184 (18)	0.0172 (17)	0.0181 (15)	−0.0027 (15)	0.0028 (13)	0.0002 (13)
C11	0.0224 (18)	0.0160 (17)	0.0147 (15)	−0.0014 (15)	0.0053 (13)	−0.0066 (12)
C12	0.0242 (19)	0.0137 (16)	0.0162 (15)	0.0012 (15)	0.0096 (13)	−0.0034 (12)
C13	0.0205 (18)	0.0113 (15)	0.0204 (16)	0.0001 (14)	0.0085 (13)	0.0027 (13)
C14	0.0217 (18)	0.0162 (17)	0.0180 (16)	−0.0017 (15)	0.0095 (14)	0.0031 (13)
C15	0.0111 (17)	0.0217 (18)	0.0237 (17)	−0.0013 (15)	0.0046 (13)	−0.0044 (14)
C16	0.0123 (17)	0.0213 (18)	0.0215 (16)	−0.0006 (15)	0.0031 (13)	−0.0016 (13)
O1W	0.0376 (17)	0.0435 (18)	0.0324 (15)	−0.0048 (15)	0.0139 (13)	0.0038 (13)

### Geometric parameters (Å, °)

**Table:**

Ru1—N1	2.088 (3)	C5—C6	1.467 (5)
Ru1—N2	2.093 (3)	C6—C7	1.392 (4)
Ru1—S3	2.2800 (9)	C7—C8	1.381 (5)
Ru1—S2	2.3012 (8)	C7—H7	0.9500
Ru1—S1	2.3120 (8)	C8—C9	1.372 (5)
Ru1—Cl1	2.4379 (8)	C8—H8	0.9500
S1—C16	1.825 (3)	C9—C10	1.382 (4)
S1—C11	1.843 (3)	C9—H9	0.9500
S2—C12	1.819 (3)	C10—H10	0.9500
S2—C13	1.836 (3)	C11—C12	1.517 (5)
S3—C14	1.825 (3)	C11—H11A	0.9900
S3—C15	1.841 (3)	C11—H11B	0.9900
N1—C1	1.350 (4)	C12—H12A	0.9900
N1—C5	1.361 (4)	C12—H12B	0.9900
N2—C10	1.343 (4)	C13—C14	1.515 (4)
N2—C6	1.361 (4)	C13—H13A	0.9900
N3—O1	1.247 (4)	C13—H13B	0.9900
N3—O3	1.247 (4)	C14—H14A	0.9900
N3—O2	1.251 (4)	C14—H14B	0.9900
C1—C2	1.378 (5)	C15—C16	1.510 (5)
C1—H1	0.9500	C15—H15A	0.9900
C2—C3	1.378 (5)	C15—H15B	0.9900
C2—H2	0.9500	C16—H16A	0.9900
C3—C4	1.385 (5)	C16—H16B	0.9900
C3—H3	0.9500	O1W—H1X	0.95 (3)
C4—C5	1.393 (4)	O1W—H1Y	0.94 (3)
C4—H4	0.9500		
			
N1—Ru1—N2	77.86 (10)	C7—C6—C5	124.1 (3)
N1—Ru1—S3	93.92 (8)	C8—C7—C6	119.8 (3)
N2—Ru1—S3	93.76 (8)	C8—C7—H7	120.1
N1—Ru1—S2	174.02 (8)	C6—C7—H7	120.1
N2—Ru1—S2	96.43 (8)	C9—C8—C7	119.2 (3)
S3—Ru1—S2	88.19 (3)	C9—C8—H8	120.4
N1—Ru1—S1	98.04 (7)	C7—C8—H8	120.4
N2—Ru1—S1	175.57 (8)	C8—C9—C10	119.0 (3)
S3—Ru1—S1	88.18 (3)	C8—C9—H9	120.5
S2—Ru1—S1	87.61 (3)	C10—C9—H9	120.5
N1—Ru1—Cl1	88.41 (8)	N2—C10—C9	122.7 (3)
N2—Ru1—Cl1	87.70 (8)	N2—C10—H10	118.6
S3—Ru1—Cl1	177.46 (3)	C9—C10—H10	118.6
S2—Ru1—Cl1	89.59 (3)	C12—C11—S1	111.4 (2)
S1—Ru1—Cl1	90.51 (3)	C12—C11—H11A	109.3
C16—S1—C11	100.05 (16)	S1—C11—H11A	109.3
C16—S1—Ru1	103.44 (11)	C12—C11—H11B	109.3
C11—S1—Ru1	106.47 (10)	S1—C11—H11B	109.3
C12—S2—C13	101.89 (15)	H11A—C11—H11B	108.0
C12—S2—Ru1	103.80 (11)	C11—C12—S2	113.2 (2)
C13—S2—Ru1	106.02 (11)	C11—C12—H12A	108.9
C14—S3—C15	99.61 (16)	S2—C12—H12A	108.9
C14—S3—Ru1	103.17 (11)	C11—C12—H12B	108.9
C15—S3—Ru1	106.61 (11)	S2—C12—H12B	108.9
C1—N1—C5	117.8 (3)	H12A—C12—H12B	107.8
C1—N1—Ru1	126.2 (2)	C14—C13—S2	110.8 (2)
C5—N1—Ru1	115.9 (2)	C14—C13—H13A	109.5
C10—N2—C6	118.5 (3)	S2—C13—H13A	109.5
C10—N2—Ru1	125.6 (2)	C14—C13—H13B	109.5
C6—N2—Ru1	115.8 (2)	S2—C13—H13B	109.5
O1—N3—O3	120.4 (3)	H13A—C13—H13B	108.1
O1—N3—O2	119.8 (3)	C13—C14—S3	112.6 (2)
O3—N3—O2	119.7 (3)	C13—C14—H14A	109.1
N1—C1—C2	122.8 (3)	S3—C14—H14A	109.1
N1—C1—H1	118.6	C13—C14—H14B	109.1
C2—C1—H1	118.6	S3—C14—H14B	109.1
C3—C2—C1	119.7 (3)	H14A—C14—H14B	107.8
C3—C2—H2	120.1	C16—C15—S3	111.5 (2)
C1—C2—H2	120.1	C16—C15—H15A	109.3
C2—C3—C4	118.3 (3)	S3—C15—H15A	109.3
C2—C3—H3	120.8	C16—C15—H15B	109.3
C4—C3—H3	120.8	S3—C15—H15B	109.3
C3—C4—C5	119.9 (3)	H15A—C15—H15B	108.0
C3—C4—H4	120.1	C15—C16—S1	113.2 (2)
C5—C4—H4	120.1	C15—C16—H16A	108.9
N1—C5—C4	121.5 (3)	S1—C16—H16A	108.9
N1—C5—C6	115.3 (3)	C15—C16—H16B	108.9
C4—C5—C6	123.2 (3)	S1—C16—H16B	108.9
N2—C6—C7	120.8 (3)	H16A—C16—H16B	107.8
N2—C6—C5	115.1 (3)	H1X—O1W—H1Y	109.6 (15)
			
N1—Ru1—S1—C16	−76.63 (14)	Ru1—N1—C1—C2	−177.6 (2)
S3—Ru1—S1—C16	17.07 (12)	N1—C1—C2—C3	1.1 (5)
S2—Ru1—S1—C16	105.33 (12)	C1—C2—C3—C4	−0.2 (5)
Cl1—Ru1—S1—C16	−165.10 (12)	C2—C3—C4—C5	−0.6 (5)
N1—Ru1—S1—C11	178.43 (14)	C1—N1—C5—C4	0.3 (4)
S3—Ru1—S1—C11	−87.87 (12)	Ru1—N1—C5—C4	177.1 (2)
S2—Ru1—S1—C11	0.39 (12)	C1—N1—C5—C6	−177.7 (3)
Cl1—Ru1—S1—C11	89.96 (12)	Ru1—N1—C5—C6	−1.0 (3)
N2—Ru1—S2—C12	−158.31 (14)	C3—C4—C5—N1	0.6 (5)
S3—Ru1—S2—C12	108.11 (12)	C3—C4—C5—C6	178.5 (3)
S1—Ru1—S2—C12	19.86 (12)	C10—N2—C6—C7	−2.2 (4)
Cl1—Ru1—S2—C12	−70.67 (12)	Ru1—N2—C6—C7	−179.6 (2)
N2—Ru1—S2—C13	94.76 (13)	C10—N2—C6—C5	176.1 (3)
S3—Ru1—S2—C13	1.18 (11)	Ru1—N2—C6—C5	−1.3 (3)
S1—Ru1—S2—C13	−87.07 (11)	N1—C5—C6—N2	1.5 (4)
Cl1—Ru1—S2—C13	−177.60 (11)	C4—C5—C6—N2	−176.5 (3)
N1—Ru1—S3—C14	−154.39 (13)	N1—C5—C6—C7	179.7 (3)
N2—Ru1—S3—C14	−76.32 (13)	C4—C5—C6—C7	1.7 (5)
S2—Ru1—S3—C14	20.01 (11)	N2—C6—C7—C8	0.6 (5)
S1—Ru1—S3—C14	107.67 (11)	C5—C6—C7—C8	−177.5 (3)
N1—Ru1—S3—C15	101.21 (14)	C6—C7—C8—C9	1.2 (5)
N2—Ru1—S3—C15	179.28 (14)	C7—C8—C9—C10	−1.3 (5)
S2—Ru1—S3—C15	−84.39 (12)	C6—N2—C10—C9	2.1 (5)
S1—Ru1—S3—C15	3.27 (12)	Ru1—N2—C10—C9	179.2 (2)
N2—Ru1—N1—C1	176.7 (3)	C8—C9—C10—N2	−0.3 (5)
S3—Ru1—N1—C1	−90.3 (3)	C16—S1—C11—C12	−133.3 (2)
S1—Ru1—N1—C1	−1.6 (3)	Ru1—S1—C11—C12	−26.0 (2)
Cl1—Ru1—N1—C1	88.7 (3)	S1—C11—C12—S2	45.9 (3)
N2—Ru1—N1—C5	0.2 (2)	C13—S2—C12—C11	67.2 (3)
S3—Ru1—N1—C5	93.2 (2)	Ru1—S2—C12—C11	−42.9 (2)
S1—Ru1—N1—C5	−178.1 (2)	C12—S2—C13—C14	−136.2 (2)
Cl1—Ru1—N1—C5	−87.8 (2)	Ru1—S2—C13—C14	−27.9 (2)
N1—Ru1—N2—C10	−176.6 (3)	S2—C13—C14—S3	48.1 (3)
S3—Ru1—N2—C10	90.2 (3)	C15—S3—C14—C13	65.5 (3)
S2—Ru1—N2—C10	1.6 (3)	Ru1—S3—C14—C13	−44.2 (3)
Cl1—Ru1—N2—C10	−87.7 (3)	C14—S3—C15—C16	−135.6 (2)
N1—Ru1—N2—C6	0.6 (2)	Ru1—S3—C15—C16	−28.6 (3)
S3—Ru1—N2—C6	−92.6 (2)	S3—C15—C16—S1	46.3 (3)
S2—Ru1—N2—C6	178.8 (2)	C11—S1—C16—C15	69.2 (3)
Cl1—Ru1—N2—C6	89.5 (2)	Ru1—S1—C16—C15	−40.6 (3)
C5—N1—C1—C2	−1.2 (5)		

### Hydrogen-bond geometry (Å, °)

**Table:**

*D*—H···*A*	*D*—H	H···*A*	*D*···*A*	*D*—H···*A*
O1W—H1X···O3^i^	0.95 (3)	2.10 (3)	3.037 (4)	169 (4)
O1W—H1Y···O2^ii^	0.94 (3)	1.92 (2)	2.839 (4)	166 (4)

Symmetry codes: (i) *x*−1, −*y*+3/2, *z*−1/2; (ii) *x*−1, *y*, *z*.
